# Tula Virus as Causative Agent of Hantavirus Disease in Immunocompetent Person, Germany

**DOI:** 10.3201/eid2704.203996

**Published:** 2021-04

**Authors:** Jörg Hofmann, Stephanie Kramer, Klaus R. Herrlinger, Kathrin Jeske, Martin Kuhns, Sabrina Weiss, Rainer G. Ulrich, Detlev H. Krüger

**Affiliations:** Institute of Virology, Charité–Universitätsmedizin Berlin, Berlin, Germany (J. Hofmann; S. Weiss, D.H. Krüger);; Asklepios Klinik Nord, Hamburg, Germany (S. Kramer, K.R. Herrlinger);; Institute of Novel and Emerging Infectious Diseases, Friedrich-Loeffler-Institut, Federal Research Institute for Animal Health, Greifswald-Insel Riems, Germany (K. Jeske, R.G. Ulrich);; Institute of Diagnostic Virology, Friedrich-Loeffler-Institut, Federal Research Institute for Animal Health, Greifswald-Insel Riems (K. Jeske);; Medilys Laborgesellschaft mbH, Hamburg (M. Kuhns)

**Keywords:** hantavirus, Tula hantavirus, renal failure, respiratory distress, viruses, respiratory infections, zoonoses, Germany, Tula virus

## Abstract

We report molecular evidence of Tula virus infection in an immunocompetent patient from Germany who had typical signs of hantavirus disease. Accumulating evidence indicates that Tula virus infection, although often considered nonpathogenic, represents a threat to human health.

Hantavirus disease, also called hemorrhagic fever with renal syndrome and hantavirus cardiopulmonary syndrome, is a zoonosis; hantaviruses are transmitted from their reservoirs (rodents) to humans. The clinical course is characterized by initial high fever and body pain, potentially proceeding to renal, pulmonary failure, or both. The case-fatality rate depends on the causal virus species and can reach up to 50% (*1*).

Tula virus (TULV), a member of the family *Hantaviridae*, genus *Orthohantavirus*, has been isolated from common voles (*Microtus arvalis*) (*2*). TULV, a broadly distributed virus in different parts of Eurasia, is hosted by common voles but has also been found in related vole species (*3*). Clinical findings of TULV pathogenicity are very rare. In 2003, a case of TULV-associated hantavirus disease was diagnosed by serologic and molecular epidemiologic means (*4*). So far, direct molecular evidence for TULV infection has only been found in 2 cases (1 in an immunocompromised patient who had severe hantavirus disease [*5*], the other in an immunocompetent person without preexisting illness who had mild hantavirus disease [*6*]).

We report molecular evidence of TULV infection in a 21-year old immunocompetent man who originated from a small village near Hamburg, northern Germany. He was admitted to hospital for sudden fever, sickness, severe headache, abdominal pain, and limb pain since the day before. His medical history was unremarkable. The patient worked as a sanitary and heating engineer in the northern part of Germany. Except for elevated body temperature, the physical examination did not reveal any abnormalities. Blood testing at the day of admission revealed thrombocytopenia (63 platelets/nL), markedly elevated C-reactive protein (63.6 mg/L), and borderline leukocyte (9.8 cells/nL) and serum creatinine (1.2 mg/dL) values. Because of biochemical signs of infection, ultrasound findings of a moderate enlargement of spleen and cervical lymph nodes as well as hints of a small lung infiltration, an antibiotic regime (ampicillin/sulbactam and clarithromycin) was initiated. Over the next few days, the fever resolved.

At day 4, biochemical signs of disturbed retention function indicated acute kidney injury. An enhanced serum creatinine value (1.6 mg/dL) and impaired (59 mL/min) glomerular filtration rate (GFR), determined using the CKD-EPI method, were noted; thrombocytopenia and an elevated C-reactive protein level persisted. The GFR, determined using the cystatin method, as measured at day 5, was reduced to 48 mL/min. The patient did not observe signs of oliguria. Under calculated intravenous fluid intake, the creatinine, GFR, and platelet values normalized until day 8, and the C-reactive protein declined to 12.1 mg/L. The patient was discharged from hospital at day 8 in good general condition.

Initial laboratory diagnostics were based on hantavirus serologic test results (on day 4 of hospitalization) using the Hantavirus Profile 1 immunoblot (Euroimmun, https://www.euroimmun.com). This assay does not contain TULV antigen, but the patient’s serum showed strong IgG reactivity with Dobrava–Belgrade virus (DOBV), Hantaan virus (HTNV), and Puumala virus (PUUV) antigens as well as strong band intensity on PUUV in the IgM assay. The recomLine HantaPlus IgG and IgM assays (Mikrogen, https://www.mikrogen.de) revealed strong IgG reactivity to PUUV and in the IgM blot strong band intensities on PUUV, Sin Nombre virus, and DOBV. On the basis of these serologic findings, a PUUV infection of the patient was suspected.

At day 5, serum was obtained for molecular virus detection. Using the primers of the Pan Hanta PCR (*7*), a 392-nt long region of the large genome segment was amplified and sequenced. The molecular phylogenetic analysis of the large segment sequence demonstrated TULV, but not PUUV, infection of the patient. Within the phylogenetic tree, the new sequence (named H18045/Winsen/19 and deposited in GenBank under accession no. MT993951) clustered with vole-derived TULV sequences from Germany and, in a more refined analysis, with those from the central north (CEN.N) clade, which consists of sequences from the northern, eastern, and central parts of the country (Figure).

**Figure:**
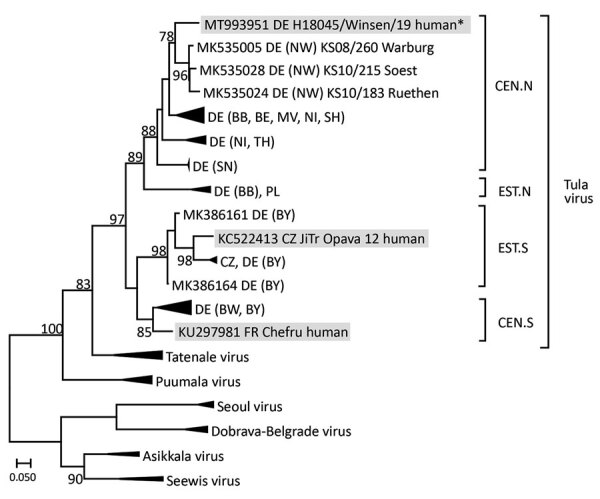
Maximum-likelihood tree of TULV from an immunocompetent patient in Germany (strain H18045/Winsen/19, marked with an asterisk [*]). Tree is based on partial large segment sequences (nucleotide position 2996–3291, according to TULV strain Moravia [GenBank accession no. NC_005226.1]). Designations of patient-derived sequences are shaded in gray. The alignment was constructed using the ClustalW Multiple Alignment algorithm implemented in Bioedit 7.2.3 (https://bioedit.software.informer.com). Maximum-likelihood analyses with 1,000 bootstraps and 50% cutoff using the general time-reversible substitution model with invariant sites and a gamma-distributed shape parameter was performed using FastTreeMP 2.1.10 (http://www.microbesonline.org) on CIPRES Science Gateway 3.3 (http://www.phylo.org). Bootstrap values >75 are given at the supported nodes. Geographic origin of TULV sequences are indicated by countries (Germany, DE; Czech Republic, CZ; France, FR; Poland, PL) and specified for Germany by adding the federal states (BE, Berlin; BB, Brandenburg; BW, Baden-Wuerttemberg, BY, Bavaria; MV, Mecklenburg-Western Pomerania, NI, Lower Saxony; NW, North Rhine-Westphalia; SH, Schleswig-Holstein; SN, Saxony; and TH, Thuringia). Triangles indicate condensed branches of TULV clades central north (CEN.N; DE: BB, GenBank accession nos. MK53017, MK53034, MK53036; BE, MK53003; MV, MK53004, MK53022; NI, MK53011, MK53032; SH, MK53033; SN HQ728453, HQ728454; TH, HQ728456, HQ728461, MK53007), eastern north (EST.N; PL: MK535037; DE: BB, MK535014–MK535015), eastern south (EST.S; CZ: MK386155–MK386156; DE: BY, MK386154, MK386161, MK386164), and central south (CEN.S; DE: BW, HQ728457, HQ728458; BY, HQ728462–HQ728464, HQ728466), as well as Puumala virus (KJ994778, MN026167, MN026168), Tatenale virus including its strain Traemmersee virus (MK542664, MK883760, MK883761, MN267824), Dobrava–Belgrade virus (JQ026206, KJ182937, KJ182938), Asikkala virus (KC880348, KC880349), Seewis virus (JQ425312, JQ425320), and Seoul virus (MG386252, KJ502300, KJ502303).

Previous studies from northeast Germany have shown that TULV is able to infect humans. Out of 563 serum samples from forest workers investigated, 22 samples (3.9%) reacted exclusively with TULV diagnostic antigen (*8*). In a survey of 6,537 serum samples representing the average population of Germany, 1 sample showed its highest neutralizing titer to TULV compared with PUUV, DOBV, HTNV, and Seoul virus (*9*).

A reason for the rare finding of TULV-associated hantavirus disease might be the close genetic and antigenic relationship to another vole-associated hantavirus, PUUV, which is carried by bank voles (*Myodes glareolus*) and is a well-known pathogenic agent broadly distributed in Europe and parts of Asia (*1*). Because anti-TULV and anti-PUUV seroreactivities cannot be distinguished using the usual serologic techniques (i.e., without neutralization assays) (*2*,*10*), TULV infections might be misdiagnosed as PUUV infections during routine diagnostics.

The ability of TULV to cause hantavirus disease even in a previously healthy person shows the pathogenic potential of this virus. Therefore, the vole species hosting TULV should be considered as infection sources in their respective geographic ranges. Because of the broad distribution (https://www.iucnredlist.org/search?query%20=%20microtus&searchType%20=%20species) and mass reproduction of common voles in several parts of Europe, TULV should be considered as a threat to human health.
